# Factors associated to hypertension knowledge and control in Kimpese, Democratic Republic of the Congo

**DOI:** 10.4102/phcfm.v17i1.4721

**Published:** 2025-03-31

**Authors:** Vainqueur N. Diakengua, Ernest K. Sumahili, Patrick N. Ntontolo, Aliocha N. Nkodila, James Ibuaku, Pieter van den Hombergh, Meena Hariharan, Louis S. Jenkins, Philippe L. Ngwala

**Affiliations:** 1 Department of Family Medicine, School of Medicine, Protestant University of Congo, Kinshasa, Democratic Republic of the Congo; 2 Department of Internal Medicine, School of Medicine, Protestant University of Congo, Kinshasa, Democratic Republic of the Congo; 3 Federal Medical Centre, Asaba, Nigeria; 4 Working Group Family Medicine and International Health Care, Hilversum, the Netherlands; 5 Centre for Health Psychology, University of Hyderabad, Hyderabad, India; 6 Department of Family Medicine, Faculty of Medicine, Stellenbosch University, Cape Town, South Africa

**Keywords:** knowledge, blood pressure control, hypertension, Kimpese, DRC

## Abstract

**Background:**

Worldwide, the proportion of hypertensive patients with controlled blood pressure is poor. Knowledge on hypertension has been recognised as a major determinant of uncontrolled hypertension.

**Aim:**

This study aimed to determine factors associated with knowledge and control of hypertension among hypertensive patients in Kimpese Health Zone, in the Democratic Republic of the Congo (DRC).

**Setting:**

Six health facilities of the Kimpese Health Zone were selected.

**Methods:**

This study was an analytical cross-sectional study from May 2021 to December 2021. Information on socio-demographic characteristics, clinical data and knowledge on hypertension was collected. Factors associated with knowledge and control of hypertension were determined using logistic regression analysis.

**Results:**

A total of 301 participants with a sex ratio of 1:3 (F > M) and a mean age of 60.5 ± 12.1 years were included in the study. Poor knowledge on hypertension (79.1%) and a treatment failure (84.3%) were common. Low educational level (*p* = 0.024; adjusted odds ratio [aOR] = 2.64 [1.72–3.73]), rural residence (*p* = 0.02; aOR = 3.34 [1.24–8.52]) and a lack of information by a health professional (physician or nurse) (*p* ≤ 0.001; aOR = 3.34 [1.24–8.52]) were significantly associated with poor knowledge. In addition, high cardiovascular risk (*p* = 0.009; aOR = 2.75 [1.29–5.84]), subclinical atherosclerosis (*p* = 0.000, AOR = 9.26 [3.54–24.23]) and absence of knowledge on hypertension (*p* = 0.042, AOR = 1.96 [1.49–2.23]) were significantly associated with uncontrolled hypertension.

**Conclusion:**

There was propensity of uncontrolled hypertension and poor knowledge among the study participants. Poor socio-demographic conditions and a lack of accurate information on hypertension increased odds of poor knowledge of the disease. In addition, insufficient knowledge on hypertension and comorbidities were associated with uncontrolled hypertension.

**Contribution:**

Education on hypertension and screening; managing comorbidities in integrating approach to non-communicable diseases are key components of managing hypertension in our setting to improve health outcomes.

## Introduction

Hypertension (HTN) is an important risk factor for cardiovascular disease, brain disease and overall mortality worldwide.^1^ The prevalence of hypertension varies across World Health Organization (WHO) regions, countries and across income levels, with the highest prevalence in Africa (27%) and the lowest in Americas (18%).^2^ In 2019, 1.28 billion people (652 million males vs. 626 million females) aged between 30 years and 79 years were affected with HTN worldwide with two-thirds living in low- and middle-income countries (LMICs).^3^ In 2016, the prevalence of hypertension in the Democratic Republic of the Congo (DRC) was estimated at 22% and the cardiovascular mortality rate at 10%.^4^

On average, 46% hypertensive patients (41% female vs. 51% male) were unaware of their condition at the time of diagnosis, and more than 56.25% of these patients were not receiving treatment. The rate of hypertension control was 23% for females and 18% for males.^3^ In sub-Saharan Africa, hypertensive patients have a high incidence of uncontrolled blood pressure.^5^ In DRC, three studies showed, respectively, 77.5%, 88.4% and 65.8% the prevalence of uncontrolled hypertension.^6,7,8^ Controlling hypertension worldwide by reducing global prevalence by 25% by 2020 is one of the targets of the WHO Global Action Plan 2013–2020 on the prevention and control of chronic non-communicable diseases (NCDs).^9^

Several factors contribute to poor HTN control: physicians-related factors, barriers in healthcare systems and patients’-related barriers. The physicians-related factors are non-adherence to treatment guidelines, failure to intensify the regimen if goals are not met and failure to emphasise therapeutic lifestyle changes. Patients-related barriers include low knowledge of hypertension, low adherence to antihypertensive treatment, false beliefs, inability to change their lifestyle, side effects of antihypertensive drugs, unrealistic expectations on antihypertensive treatment, socio-demographic factors,^10^ secondary hypertension,^11^ co-morbidity of hypertension, female gender^12^ and diabetes mellitus.^13^ Kika et al. found in Kinshasa health centres that the main factors associated with the non-control of hypertension were metabolic syndrome and non-compliance with antihypertensive treatment.^6^

Hypertensive patients’ knowledge on their disease is associated with adherence to treatment and consequently with blood pressure control.^14^ It is therefore one of the most important patient-related factors influencing HTN control alongside attitude, social support, stress and treatment complexity.^15^ Several factors can influence hypertensive patients’ knowledge and include age, family history of hypertension, body mass index (BMI), education level^16^; gender, years of education, duration of hypertension since diagnosis,^17^ marital status, income level and physical activity.^18^

In Kinshasa, it was found that patients with hypertension had little knowledge of their condition in terms of risk factors, clinical manifestations and complications: Anxiety was recognised as the main risk factor at 67.6%, while salt consumption, alcohol intake and other factors accounted for 6.3%, 4.8% and 7.1% of responses, respectively. On the other hand, 15.4% did not know any risk factor for hypertension. With regard to complications of hypertension, stroke and death accounted for 37.2% and 27.8%, respectively, while diabetes, cardiovascular disease and others accounted for 5.6%, 3.5% and 4.6% of the respondents.^7^

This study aimed to determine factors associated with the knowledge and control of hypertension. The objectives of the study being: to describe socio-demographic characteristics, clinical data and the treatment failure among hypertensive patients; to assess the level of knowledge of hypertensive patients attending health facilities; to determine factors associated with the knowledge of hypertension and to determine factors associated with the control of hypertension.

The expanded chronic care model (ECCM) framework was used to address this question. It is an enhanced version of chronic care model (CCM) where improved functional and clinical outcomes for disease management are the result of productive interactions between Informed, Activated Patient and the prepared, proactive practice team of clinicians and healthcare professionals with the integration of prevention and population health promotion in order to develop the community portion of the CCM.^19^

## Methods

### Study design

This was a cross-sectional study.

### Setting

The study was conducted in six health facilities of the city of Kimpese in Kimpese Health Zone (KHZ), Province of Kongo Central in DRC, from May 2021 to December 2021. Kimpese is a city located at 220 km, west of Kinshasa, the capital of the DRC with around 100 000 inhabitants and belonging to the rural KHZ. The KHZ is serving 187 796 people and is divided into 20 health areas. Kimpese is the biggest city of KHZ including five health areas.

The health facilities included in the study were the General Reference Hospital of IME (GRHI), a faith-based hospital and five health facilities: Lamba, Ceco, La Famille, Nkebolo and Christ-Vie. The other five health facilities are health centres offering minimal package activities (MPAs) to the population including promotion, preventive and basic curative care.

### Study population

The study population consisted of all hypertensive patients seen in outpatient services in the six selected facilities. The inclusion criteria were age over 18 years, registered hypertensive patients receiving antihypertensive treatment for more than 1 month before the study and freely consenting to participate in the study.

### Sampling

The sample size was calculated using Fischer’s formula: *n* ≥ (Z2 × (*p*) (1−*p*))/*d*^2^ with the following assumptions *z* = 1.96 (confidence coefficient), *p* = 0.225 (prevalence of uncontrolled hypertension in Kinshasa)^6^ as the *d* = 0.05 (margin of error or range of inaccuracies reflecting the desired degree of absolute precision). The sample size was calculated as *n* ≥ (1.96)^2^ × 0.225 × 0.7755/ (0.05)^2^ = 268. Incorporating the 10% of non-responders, a sample of ≥ 295 participants was obtained. A non-probability sampling was used: all the patients attending the outpatient services on the day of the recruitment were invited to enter in the study until the desired sample size was reached. The choice of this recruitment method was made because of financial and time constraints.

### Data collection

The patients were recruited in the six health facilities after the outpatient routine consultation. Patients seen during the routine visit were invited to participate in the study once a week in each health centre. We advised consultants to direct all hypertensive patients to the principal investigator. After the consent of the participants has been obtained, the blood pressure was measured along with their relevant clinical parameters. Finally, the questionnaire was administered to the participants.

The data were collected using a structured questionnaire by the principal investigator and two trained physicians after conducting a pre-test.

The questionnaire was written in French and then translated into Kikongo by linguistic experts from the Institut Supérieur Pédagogique de Mbanza Ngungu. The two versions (French and Kikongo) were translated back to English to verify the conformity and the corrections made if necessary. It consisted of three parts: the first part included socio-demographic variables (age, sex, place of residency, educational level, marital status, professional occupation and religion); the second part of the questionnaire included clinical data such as weight, systolic and diastolic blood pressure (DBP), pulse pressure (PP), mean arterial pressure (MAP), height, co-morbidities (diabetes mellitus, obesity, subclinical atherosclerosis), lifestyle (smoking, alcohol consumption, exercise, salt intake), waist circumference (WC) and cardiovascular risk (CVR). The third part of the questionnaire included the level of knowledge about hypertension using a validated test (Cronbach’s alpha of 0.92)^20^ translated from English to French and Kikongo.

### Operational definitions and measurements

The height, weight and blood pressure were measured according to the recommendations from the WHO STEPwise approach to NCD risk factor surveillance.^21^ The blood pressure was measured with an OMRONR MIT5 electronic blood pressure monitor (HEM-7280T-E). The average of the three blood pressure measurements was recorded for the participant.^22^ A SECA scale of 260 was used to assess the weight of the participants.

In this study, uncontrolled hypertension was defined as systolic blood pressure (SBP) ≥ 140 mm Hg and DBP ≥ 90 mm Hg.^23^

The PP was calculated as systolic minus DBP; subclinical atherosclerosis was defined as PP > 60 mmHg.

The BMI was calculated as weight (kg)/height (squared metres) and was categorised as follows: underweight (BMI < 18.5 kg/m^2^), normal weighted (18.55 kg/m^2^ – 24.99 kg/m^2^), overweight (BMI ≥ 25 kg/m^2^) and obese ≥ 30 kg/m^2^.^24^ For the WC, normal values were defined according to sex: males ˂ 102 cm and females ˂ 88 cm.^25^

The knowledge on hypertension was assessed using the hypertension knowledge test scale. In univariate analysis, three categories were considered: low (score: −10), medium (score: 11–16) and high (score: 17–22). For the multivariate analysis, as dependent variable, two categories were considered, poor level of knowledge (score: 0–10) and high level of knowledge (score: 11–22).

The participants were asked to report if yes or not, they were using consuming alcohol or smoking or taking excessive salt (more than 1 teaspoon/day^26^ or doing physical exercise at least three times weekly of at least 30 min daily).^27^

### Data analysis

Descriptive statistics included means ± standard deviations or medians (Interquartile range [IQR]) for normally distributed and non-normally distributed quantitative variables. The proportions were used to summarise categorical variables. Comparison of categorical variables with regard to the control of hypertension or the knowledge was performed using chi-square and Fisher’s exact test. Multivariate logistic regression was used to identify independent factors associated with knowledge of hypertension or control of hypertension. The value of *p* < 0.05 was the threshold of significance retained.

### Ethical considerations

The study was conducted following the Declaration of Helsinki and the National Human Research Ethics Guidelines. It was approved by the Ethics Committee of the Protestant University of Congo (Ref. CEUPC 0068).

## Results

A total of301 hypertensive patients were recruited in the study, respectively, from GRHI (66.1%), La Famille (7%), Chist-Vie (5%), Nkebolo (6.3%), Ceco (10%) and Lamba (5.6%ba). Table 1 summarises socio-demographic characteristics of the participants. Male participants differed from the female participants by marital status (*p* < 0.001), profession (*p* < 0.001), educational level (*p* < 0.001) and place of residence (*p* = 0.011).

**TABLE 1:** Socio-demographics of the participants with hypertension at Kimpese Health Zone, 2021 (*N* = 301).

Variables	All (*N* = 301)		Male (*n* = 129)		Female (*n* = 172)		*p*-value
	*N*	%	*n*	%	*n*	%	
**Age range (years)**	-	-	-	-	-	-	0.528
< 50	56	18.6	22	17.1	34	19.8	-
50–59	79	26.2	30	23.3	49	28.5	-
60–69	95	31.6	46	35.7	49	28.5	-
≥ 70	71	23.6	31	24.0	40	23.3	-
**Marital status**	-	-	-	-	-	-	< 0.001
Married	193	64.1	102	79.1	91	52.9	-
Unmarried	108	35.9	27	20.9	81	47.1	-
**Profession**	-	-	-	-	-	-	< 0.001
Formal	77	25.6	51	39.5	26	15.1	-
Without profession	95	31.6	43	33.3	52	30.2	-
Informal	129	42.9	35	27.1	94	54.7	-
**Educational level**	-	-	-	-	-	-	< 0.001
Without	21	7.0	1	0.8	20	11.6	-
Primary	82	27.2	24	18.6	58	33.7	-
Secondary	159	52.8	75	58.1	84	48.8	-
University	39	13.0	29	22.5	10	5.8	-
**Place of residence**	-	-	-	-	-	-	0.011
Rural	35	11.6	22	17.1	13	7.6	-
Urban	73	24.3	35	27.1	38	22.1	-
Semi-urban	193	64.1	72	55.8	121	70.3	-
**Religion**	-	-	-	-	-	-	0.117
Catholic	93	30.9	32	24.8	61	35.5	-
Protestant	106	35.2	45	34.9	61	35.5	-
Kimbanguist	36	12.0	20	15.5	16	9.3	-
Rival church	66	21.9	32	24.8	34	19.8	-

Note: Sex ratio = 1/1.3; Mean age (years): All = 60.5 ± 12.1; Male = 61.3 ± 11.8; Female = 59.9 ± 12.3.

With regard to alcohol consumption (41.1%; *p* < 0.001), smoking (14.7%; *p* < 0.001), physical activity (52.7%; *p* = 0.005), excessive salt intake (27.1%; *p* = 0.030), knowledge of hypertension as an aggravating factor of coronavirus disease 2019 (COVID-19) infection (30.2%; *p* = 0.003) and high CVR (69.8%, *p* < 0.001), males were significantly different and higher than females. On the other hand, the proportions of general obesity (27.9%, *p* < 0.001) and abdominal obesity (76.2%; *p* < 0.001) in females were significantly higher than in males (Table 2).

**TABLE 2:** Clinical parameters of participants with hypertension at Kimpese Health Zone, 2021 (*N* = 301).

Variables	All (*N* = 301)		Male (*n* = 129)		Female (*n* = 172)		*p*
	*N*	%	*n*	%	*n*	%	
Alcohol consumption	63	20.9	53	41.1	10	5.8	< 0.001
Smoking	25	8.3	19	14.7	6	3.5	< 0.001
Physical activity	132	43.9	68	52.7	64	37.2	0.005
Excessive salt intake	65	21.6	35	27.1	30	17.4	0.030
Knowing hypertension as an aggravating factor of COVID-19 infection	67	22.3	39	30.2	28	16.3	0.003
CVR (%)	29.2	26.3–31.0	35.2	30.4–38.7	25.2	20.9–28.4	< 0.001
Overall obesity (BMI)	59	19.6	11	8.5	48	27.9	< 0.001

Note: Abdominal obesity (WC): All = 90.9 ± 14.8; Male = 86.9 ± 12.5; Female = 94.0 ± 15.7; *p* ≤ 0.001.

COVID-19, coronavirus disease 2019; CVR, cardiovascular risk; BMI, body mass index; WC, waist circumference.

### Knowledge about hypertension

**FIGURE 1:**
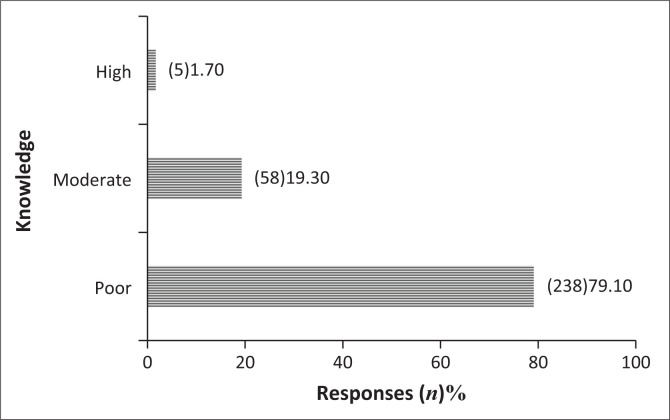
Global hypertension knowledge.

The majority of the participants had inadequate knowledge about hypertension (79.1%) (Figure 1).

**FIGURE 2:**
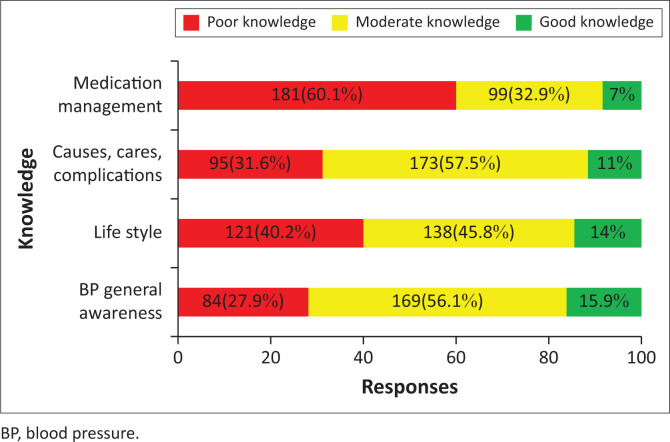
Specific areas of knowledge on hypertension among participants with hypertension, *n* = 301, 2021.

The level of knowledge on specific areas of knowledge on hypertension is displayed in Figure 2. Most of the participants expressed poor level on the medication management of hypertension (60.1%).

Regarding the knowledge of hypertension, only a low level of education, rural residence, knowledge of hypertension as an aggravating factor of COVID-19 and lack of information on hypertension by a health care provider (doctor or nurse) remained significantly associated overall poor knowledge on hypertension (Table 3).

**TABLE 3:** Factors associated with poor overall knowledge of hypertension.

Variables	Univariate analysis			Multivariate analysis		
	*p*-value	OR	95% CI	*p*-value	aOR	95% CI
**Gender**						
Male	-	1.00	-	-	1.00	-
Female	0.011	2.08	1.18–3.65	0.163	1.62	0.82–3.17
**Educational level**						
High (secondary-university)	-	1.00	-	-	1.00	-
Low (non-primary)	0.002	2.98	1.48–6.00	0.024	2.64	1.72–3.73
**Place of residence**						
Urban	-	1.00	-	-	1.00	-
Semi-urban	0.115	0.31	0.07–1.34	0.117	0.58	0.26–1.76
Rural	< 0.001	3.32	1.81–6.06	0.026	3.34	1.24–8.52
**Diabetes mellitus**						
Yes	-	1.00	-	-	1.00	-
No	0.048	1.71	1.23–3.15	0.311	1.44	0.71–2.92
**Know HBP as aggravating factor of COVID-19**						
Yes	-	1.00	-	-	1.00	-
No	< 0.001	5.99	3.25–11.02	0.001	3.35	1.67–6.75
**HBP information by a health professional**						
Yes	-	1.00	-	-	1.00	-
No	< 0.001	4.66	2.50–8.70	< 0.001	3.57	1.82–6.98

OR, odds ratio; aOR, adjusted odds ratio; CI, confidence interval; HBP, high blood pressure.

### Hypertension control

Of the 301 hypertensives, 287 or 95.3% were on antihypertensive treatment of which only 15.7% had controlled hypertension while 84.3% were uncontrolled. Among those uncontrolled 78.5% had both systolic and diastolic failure on treatment, while 18.2% had systolic uncontrol and 3.3% had diastolic uncontrol. The proportion of participants with uncontrolled hypertension was higher at GRHI (62.8%) than in other health facilities. In multivariate analysis, high CVR, presence of subclinical arteriosclerosis and lack of knowledge about hypertension increased the odds of uncontrolled hypertension by, respectively, of 3, 9 and 2 times (Table 4).

**TABLE 4:** Factors associated with uncontrolled hypertension.

Factors	Univariate analysis			Multivariate analysis		
	*p*-value	OR	95% CI	*p*-value	aOR	95% CI
**Cardiovascular risk**						
Low	-	1.00	-	-	1.00	-
Moderate	0.005	2.79	1.36–5.76	0.318	1.46	0.69–3.07
High	< 0.001	5.05	2.46–40.38	0.009	2.75	1.29–5.84
**Overweight**						
No	-	1.00	-	-	1.00	-
Yes	0.046	1.77	1.09–3.22	0.375	1.33	0.71–2.48
**Subclinical atherosclerosis (PP > 60 mmhg)**						
No	-	1.00	-	-	1.00	-
Yes	< 0.001	11.60	4.56–29.48	0.000	9.26	3.54–24.23
**Global knowledge on blood pressure**						
Yes	-	1.00	-	-	1.00	-
No	0.034	2.79	1.38–4.65	0.042	1.96	1.49–2.23

OR, odds ratio; aOR, adjusted odds ratio; CI, confidence interval; PP, pulse pressure.

## Discussion

This study focused on determining associated factors of knowledge and hypertension control among hypertensive patients, followed in outpatient clinic at six primary healthcare facilities in the city of Kimpese, HZK. Low educational level, rural residence, hypertension knowledge as a COVID-19 risk factor and a lack of information on hypertension by a health professional (doctor or nurse) were associated with poor knowledge. The factors associated with hypertension control included high CVR, the presence of subclinical arteriosclerosis and a lack of knowledge about HTN.

As found in our study, the female preponderance has also been reported in other studies, notably in Angola, Ethiopia, South Africa and even in the DRC capital city.^28,29,30,31^ This could be explained in part by the mean age of our study population (60.5 ± 12.1 years). It has been described that the frequency of hypertension in males predominates that of females before the age of 60 years, but after this age, hypertension is predominated in females following the menopause.^32^ However, another study carried out in Kinshasa found a male predominance linked to his study population, the military, where more males than females are enlisted.^33^

The high proportion of participants with low level of knowledge found in our study was corroborated by findings from other studies in the DRC^34,35^ and elsewhere.^14,16,18,36,37^ In the previous study in Kinshasa, DRC low knowledge was associated with increased CVR among hypertensive patients, thus appealing for strategies and education programmes to influence positively their attitudes and practices.^35^ Living in a rural or remote environment presents unique challenges for people with chronic conditions, mainly those created by limited healthcare services, physical and emotional isolation, limited access public or reliable transportation and cost associated.^38,39^ Low educational level could impact on the ability of the patient to conceptualise the disease in an accurate manner and contribute to low knowledge on the disease.^40^

Less than 13% of the participants had their blood pressure controlled. In our study, there was no association between the hypertension control and the socio-demographic characteristics of the participants; this corroborated the findings of other studies.^16,37,41,42,43^ Our study found an association between the lack of hypertension knowledge and non-control of hypertension as demonstrated by several other previous studies in other rural settings in DRC^34^ and other studies around the world: Pakistan,^14^ Turkey^33^ and Rwanda.^44^ Hypertension often coexists with co-morbidities^45^; our study showed that no control of hypertension was associated with patients with high CVR and those with subclinical atherosclerosis. Coexisting comorbidities complicate the management of hypertension leading to uncontrolled blood pressure.^46^ The treatment of hypertension must form part of an integrated approach to the management of non-transmissible diseases, enabling comorbidities to be identified and treated with a view to achieving better care outcomes.

### Limitations and strengths

This study was limited to anamnestic and clinical aspects associated with knowledge and control of hypertension, while paraclinical aspects such as level of cholesterol, urea, creatinine and blood glucose measurement were beyond our budget. The study population was only based on the hypertensive outpatients visiting health facilities, and results could not be generalised to all hypertensive patients, because of the non-probability sampling technique used which introduced potential selection bias. This choice was made because of time and financial resource limitations. The self-reporting of knowledge by the participants is a potential source of information bias. The translated knowledge questionnaire has not undergone the full process of cross-cultural adaptation and validation. As a result, it cannot be considered a standard tool for routinely assessing hypertension knowledge in our setting. Strengths were that it was the first study in our area to have evaluated the factors associated with uncontrolled hypertension in hypertensive patients followed in the health facilities in the city of Kimpese, HZK. The results will contribute to the improvement of the management of hypertension in this city. The study provided information that could be used in intervention in health education for hypertensive patients.

## Conclusion

The study demonstrates that hypertensive patients in our health facilities have poor overall hypertension knowledge, which is associated with non-control of hypertension and confirms high levels of uncontrolled hypertension and poor disease knowledge in a rural setting in DRC. The picture that emerges is of patients with low health literacy on hypertension and its treatment, leading to low or no compliance, continuation of an unhealthy lifestyle. The lack of appropriate information about the disease from healthcare professionals is one of the major factors. These results highlight the necessity of acting effectively to improve hypertension control and prevent adverse outcomes in rural settings because current strategy of hypertension management is focused on hospital based. Among the strategies to set a tailored educational programme on high blood pressure (HBP) both from patient and health care provider (HCP) is needed. The adoption of the nationalpolicy and real integration of NCD management in the health system based on the primary healthcare is crucial. Several guidelines by WHO and scientific societies are available and must be adapted in our context.^42,43^ Therefore, it is crucial to improve government commitment in order to increase screening, patient awareness, access to medicines and treatment control by designing country-specific programmes.

Future research to explore the impact of task shifting in the management of hypertension rural settings and impact of sociocultural factors and interaction of hypertension with other comorbidities are welcome.
